# Supplementary material for: Evaluating supply chain management of SARS-CoV-2 point-of-care (POC) diagnostic services in primary healthcare clinics in Mopani District, Limpopo Province, South Africa
Source: PLoS One. 2023 Jun 27;18(6):e0287477. doi: 10.1371/journal.pone.0287477 (PMC10298766; doi:10.1371/journal.pone.0287477)
Supplement: S1 Fig — (DOCX) [file pone.0287477.s001.docx]

**Supply chain management audit tool**

**Developing a novel approach for improving supply chain management for SARS-CoV-2 point of care diagnostic services in resource-limited settings using Mopani District in Limpopo province, South Africa as a case study**

| **Name of clinic** |  |
| --- | --- |
| **Annual headcount** |  |
| **Ideal clinic score** |  |
| **Name of Sub-district** |  |
| **Occupation of assessor** |  |
| **Role of assessor in SCM** |  |
| **Date** |  |

| **Selection** | **Yes** | **No** | **N/A** | **Comment** |
| --- | --- | --- | --- | --- |
| 1. Are managers/relevant persons responsible for SARS-CoV-2 POC diagnostic selection for the facility? |  |  |  |  |
| 1. Are these diagnostics sensitive with very few false negatives? |  |  |  |  |
| 1. Are these diagnostics specific with very few false positives? |  |  |  |  |
| 1. Are existing SARS-CoV-2 POC diagnostics user –friendly (simple to perform and requiring minimal training)? |  |  |  |  |
| 1. Do existing SARS-CoV-2 POC diagnostics enable rapid testing and treatment rapid at first visit? |  |  |  |  |
| 1. Are these diagnostics robust, for example not requiring refrigerated storage |  |  |  |  |
| 1. Are existing SARS-CoV-2 POC diagnostics equipment-free? |  |  |  |  |
| 1. Are these SARS-CoV-2 POC diagnostics delivered to those who need it? |  |  |  |  |
| **Quantification** | **yes** | **No** | **N/A** | **Comment** |
| 1. Do managers/relevant persons forecast by predicting demand based on seasonal variations (COVId-19 waves)? |  |  |  |  |
| **Storage** | **Yes** | **No** | **N/A** | **Comment** |
| 1. Does a storeroom for SARS-CoV-2 POC diagnostics exist? |  |  |  |  |
| 1. Does the SARS-CoV-2 POC diagnostic have special storage requirements? |  |  |  |  |
| 1. If yes, are storage facilities available? |  |  |  |  |
| 1. Availability of storage conditions such as light, temperature, and sanitation, for test kits and diagnostic reagents |  |  |  |  |
| **Inventory management** | **Yes** | **No** | **N/A** | **Comment** |
| 1. Availability of personnel whose duties include management of existing SARS-CoV-2 POC diagnostics at the facility? |  |  |  |  |
| 1. Presence of updated list of existing SARS-CoV-2 POC diagnostics in the last three months? |  |  |  |  |
| 1. Document expiring dates of existing SARS-CoV-2 POC diagnostics |  |  |  |  |
| 1. Document inventory levels for SARS-CoV-2 POC diagnostics |  |  |  |  |
| 1. Document unexplained losses (leakage) of SARS-CoV-2 POC diagnostics |  |  |  |  |
| 1. Availability of computerized recorded inventory (stock visibility system) |  |  |  |  |
| 1. Availability of manual recorded inventory |  |  |  |  |
| 1. Availability of basic records cards such as stock or bin cards |  |  |  |  |
| 1. Availability of monthly consumption records |  |  |  |  |
| 1. Availability of inventory control forms |  |  |  |  |
| 1. Availability of expired SARS-CoV-2 POC diagnostics |  |  |  |  |
| 1. Compile list of expired SARS-CoV-2 POC diagnostics |  |  |  |  |
| **Procurement** | **Yes** | **No** | **N/A** | **Comment** |
| 1. How often is existing SARS-CoV-2 POC diagnostics requisition made? (Please choose only one option) |  |  |  |  |
| 1. Daily |  |  |  |  |
| 1. Weekly |  |  |  |  |
| 1. Monthly |  |  |  |  |
| 1. Quarterly |  |  |  |  |
| 1. Every 6 months |  |  |  |  |
| 1. Annually |  |  |  |  |
| 1. Per demand |  |  |  |  |
| 1. How often are existing POC diagnostics supplied following requisition? (Please choose only one) |  |  |  |  |
| 1. Daily |  |  |  |  |
| 1. Weekly |  |  |  |  |
| 1. Monthly |  |  |  |  |
| 1. Quarterly |  |  |  |  |
| 1. Every 6 months |  |  |  |  |
| 1. Annually |  |  |  |  |
| **Distribution** | **Yes** | **No** | **N/A** | **Comment** |
| 1. Check the delivery form that came with the supplies? |  |  |  |  |
| 1. Check the supplies against the delivery form and the requisition book? |  |  |  |  |
| 1. Ask the driver or delivery person to note any difference? |  |  |  |  |
| 1. Ask the delivery person to sign the accompany form before leaving your facility? |  |  |  |  |
| 1. Write down delivery information in a ledger book? |  |  |  |  |
| 1. Keep all delivery forms in a safe place? |  |  |  |  |
| 1. Document all differences? |  |  |  |  |
| **Redistribution** | **Yes** | **No** | **N/A** | **Comment** |
| 1. Are there procedures in place to redistribute SARS-CoV-2 POC diagnostics to other facilities when expiry date is close? |  |  |  |  |
| **Quality Assurance** | **Yes** | **No** | **N/A** | **Comment** |
| 1. Is the box containing SARS-CoV-2 POC diagnostics sealed upon delivery? |  |  |  |  |
| 1. Are the individual SARS-CoV-2 POC diagnostics sealed upon delivery? |  |  |  |  |
| **Human Resource Capacity** | **Yes** | **No** | **N/A** | **Comment** |
| 1. Are users trained to use existing SARS-CoV-2 POC diagnostics appropriately? |  |  |  |  |
| 1. Do they need training updates for SARS-CoV-2 POC testing or procedures? |  |  |  |  |
| 1. Are approved, written standard operating procedures (SOP) available for performing each SARS-CoV-2 POC test? |  |  |  |  |
| 1. Availability SOP available for stock (Reagents) level management for existing SARS-CoV-2 POC diagnostics? |  |  |  |  |
| 1. Does an SOP available for safe disposal of existing SARS-CoV-2 POC diagnostics? |  |  |  |  |

**Thank for taking time to complete this questionnaire.**
