# Supplementary material for: Evaluating supply chain management of SARS-CoV-2 point-of-care (POC) diagnostic services in primary healthcare clinics in Mopani District, Limpopo Province, South Africa
Source: PLoS One. 2023 Jun 27;18(6):e0287477. doi: 10.1371/journal.pone.0287477 (PMC10298766; doi:10.1371/journal.pone.0287477)
Supplement: S1 Table — (DOCX) [file pone.0287477.s002.docx]

Supplementary material 1: Characteristics of the 47 participating PHC clinics in Mopani district

| **Name of Clinic** | **Sub-district** | **Annual Head**  **count** | **Ideal clinic score** | **SCM Audit Components** | | | | | | | | | **SCM compliance rating (%)** |
| --- | --- | --- | --- | --- | --- | --- | --- | --- | --- | --- | --- | --- | --- |
|  |  |  |  | **Selection** | **Quantification** | **Storage** | **Inventory Management** | **procurement** | **Distribution** | **Redistribution** | **Quality assurance** | **Human resource** |  |
| **Basani** | Greater Giyani | 38671 | 76 | 7 (87.5) | 1 (100) | 4 (100) | 7 (58.33) | 2 (100) | 3 (42.86) | 1 (100) | 2 (100) | 2 (40) | 80.96556 |
| **Hlaneki** | Greater Giyani | 22651 | 66 | 7 (87.5) | 0 (0) | 4 (100) | 7 (58.33) | 2 (100) | 3 (42.86) | 1 (100) | 2 (100) | 5 (100) | 76.52111 |
| **Ndengeza** | Greater Giyani | 19906 | 61 | 7 (87.5) | 1 (100) | 4 (100) | 1 (8.33) | 2 (100) | 4 (57.14) | 1 (100) | 2 (100) | 2 (40) | 76.99667 |
| **Nkhensani Gateway** | Greater Giyani | 18025 | 80 | 7 (87.5) | 1 (100) | 4 (100) | 7 (58.33) | 2 (100) | 3 (42.86) | 1 (100) | 2 (100) | 2 (40) | 80.96556 |
| **Dzumeri** | Greater Giyani | 52697 | 65 | 7 (87.5) | 1 (100) | 4 (100) | 7 (58.33) | 2 (100) | 4 (57.14) | 1 (100) | 2 (100) | 2 (40) | 82.55222 |
| **Kheyi** | Greater Giyani | 13431 | 59 | 7 (87.5) | 0 (0) | 2 (50) | 7 (58.33) | 2 (100) | 3 (42.86) | 1 (100) | 2 (100) | 1 (20) | 62.07667 |
| **Loloka** | Greater Giyani | 17305 | 88 | 7 (87.5) | 1 (100) | 4 (100) | 7 (58.33) | 2 (100) | 1 (14.29) | 1 (100) | 2 (100) | 2 (40) | 77.79111 |
| **Makhuva** | Greater Giyani | 27130 | 40 | 7 (87.5) | 0 (0) | 3 (75) | 7 (58.33) | 2 (100) | 3 (42.86) | 1 (100) | 2 (100) | 2 (40) | 67.07667 |
| **Giyani CHC** | Greater Giyani | 78036 | 62 | 7 (87.5) | 1 (100) | 4 (100) | 7 (58.33) | 2 (100) | 4 (57.14) | 1 (100) | 2 (100) | 5 (100) | 89.21889 |
| **Thomo** | Greater Giyani | 22485 | 78 | 7 (87.5) | 1 (100) | 4 (100) | 7 (58.33) | 2 (100) | 4 (57.14) | 1 (100) | 2 (100) | 2 (40) | 82.55222 |
| **Kremetart** | Greater Giyani | 53813 | 39 | 7 (87.5) | 1 (100) | 4 (100) | 7 (58.33) | 2 (100) | 1 (14.29) | 1 (100) | 2 (100) | 2 (40) | 77.79111 |
| **Ngove** | Greater Giyani | 28970 | 63 | 7 (87.5) | 1 (100) | 4 (100) | 7 (58.33) | 2 (100) | 2 (28.57) | 1 (100) | 2 (100) | 1 (20) | 77.15556 |
| **Nkomo** | Greater Giyani | 25277 | 64 | 7 (87.5) | 0 (0) | 4 (100) | 2 (16.67) | 2 (100) | 3 (42.86) | 1 (100) | 2 (100) | 2 (40) | 65.22556 |
| **Shikhumba** | Greater Giyani | 21401 | 57 | 7 (87.5) | 0 (0) | 4 (100) | 1 (8.33) | 2 (100) | 2 (28.57) | 1 (100) | 2 (100) | 1 (20) | 60.48889 |
| **Lulekani** | Ba-Phalaborwa | 56461 | 93 | 7 (87.5) | 1 (100) | 4 (100) | 7 (58.33) | 2 (100) | 4 (57.14) | 1 (100) | 2 (100) | 3 (60) | 84.77444 |
| **Namakgale A** | Ba-Phalaborwa | 23457 | 54 | 7 (87.5) | 1 (100) | 4 (100) | 7 (58.33) | 2 (100) | 3 (42.86) | 1 (100) | 2 (100) | 2 (40) | 80.96556 |
| **Makhushane** | Ba-Phalaborwa | 29921 | 70 | 7 (87.5) | 1 (100) | 4 (100) | 7 (58.33) | 2 (100) | 3 (42.86) | 1 (100) | 2 (100) | 2 (40) | 80.96556 |
| **Seloane** | Ba-Phalaborwa | 17604 | 91 | 7 (87.5) | 1 (100) | 4 (100) | 7 (58.33) | 2 (100) | 3 (42.86) | 1 (100) | 2 (100) | 2 (40) | 80.96556 |
| **Ben Farm** | Ba-Phalaborwa | 31554 | 83 | 7 (87.5) | 1 (100) | 4 (100) | 7 (58.33) | 2 (100) | 4 (57.14) | 1 (100) | 2 (100) | 2 (40) | 82.55222 |
| **Phalaborwa Busstop** | Ba-Phalaborwa | 22630 | 95 | 7 (87.5) | 1 (100) | 4 (100) | 7 (58.33) | 2 (100) | 4 (57.14) | 1 (100) | 2 (100) | 2 (40) | 82.55222 |
| **Hoedsprit** | Maruleng | 19777 | 79 | 7 (87.5) | 1 (100) | 4 (100) | 7 (58.33) | 2 (100) | 3 (42.86) | 1 (100) | 2 (100) | 2 (40) | 80.96556 |
| **Lorraine** | Maruleng | 20577 | 71 | 7 (87.5) | 1 (100) | 3 (75) | 7 (58.33) | 2 (100) | 3 (42.86) | 1 (100) | 2 (100) | 2 (40) | 78.18778 |
| **Sekororo Gateway** | Maruleng | 24338 | 60 | 7 (87.5) | 1 (100) | 4 (100) | 7 (58.33) | 2 (100) | 0 (0) | 1 (100) | 2 (100) | 5 (100) | 82.87 |
| **Sekororo** | Maruleng | 21880 | 85 | 7 (87.5) | 1 (100) | 4 (100) | 7 (58.33) | 2 (100) | 4 (57.14) | 1 (100) | 2 (100) | 2 (40) | 82.55222 |
| **The Oaks** | Maruleng | 20746 | 66 | 7 (87.5) | 1 (100) | 4 (100) | 8 (66.67) | 2 (100) | 5 (71.43) | 1 (100) | 2 (100) | 2 (40) | 85.06667 |
| **Turkey** | Maruleng | 37707 | 52 | 7 (87.5) | 1 (100) | 4 (100) | 7 (58.33) | 2 (100) | 4 (57.14) | 1 (100) | 2 (100) | 2 (40) | 82.55222 |
| **Willows** | Maruleng | 32896 | 61 | 7 (87.5) | 1 (100) | 1 (25) | 7 (58.33) | 2 (100) | 4 (57.14) | 1 (100) | 2 (100) | 2 (40) | 74.21889 |
| **Bolebedu** | Greater Letaba | 18657 | 71 | 7 (87.5) | 1 (100) | 4 (100) | 7 (58.33) | 2 (100) | 4 (57.14) | 1 (100) | 2 (100) | 2 (40) | 82.55222 |
| **Modjadji V** | Greater Letaba | 19827 | 78 | 7 (87.5) | 1 (100) | 2 (50) | 7 (58.33) | 2 (100) | 3 (42.86) | 1 (100) | 2 (100) | 2 (40) | 75.41 |
| **Maphalle** | Greater Letaba | 32370 | 75 | 7 (87.5) | 1 (100) | 4 (100) | 1 (8.33) | 2 (100) | 3 (42.86) | 1 (100) | 2 (100) | 2 (40) | 75.41 |
| **Lebaka** | Greater Letaba | 25811 | 79 | 7 (87.5) | 1 (100) | 4 (100) | 7 (58.33) | 2 (100) | 4 (57.14) | 1 (100) | 2 (100) | 2 (40) | 82.55222 |
| **Bellevue** | Greater Letaba | 31173 | 57 | 7 (87.5) | 1 (100) | 4 (100) | 7 (58.33) | 2 (100) | 4 (57.14) | 1 (100) | 2 (100) | 2 (40) | 82.55222 |
| **Rotterdam** | Greater Letaba | 17436 | 51 | 7 (87.5) | 1 (100) | 4 (100) | 7 (58.33) | 2 (100) | 4 (57.14) | 1 (100) | 2 (100) | 2 (40) | 82.55222 |
| **Middelwater** | Greater Letaba | 27708 | 69 | 7 (87.5) | 1 (100) | 4 (100) | 7 (58.33) | 2 (100) | 4 (57.14) | 1 (100) | 2 (100) | 2 (40) | 82.55222 |
| **Duiwelskloof** | Greater Letaba | 24592 | 44 | 7 (87.5) | 1 (100) | 4 (100) | 0 (0) | 2 (100) | 2 (28.57) | 1 (100) | 2 (100) | 2 (40) | 72.89667 |
| **Kgapane** | Greater Letaba | 36750 | 91 | 7 (87.5) | 1 (100) | 4 (100) | 7 (58.33) | 2 (100) | 4 (57.14) | 1 (100) | 2 (100) | 2 (40) | 82.55222 |
| **Medigen** | Greater Letaba | 27520 | 84 | 7 (87.5) | 1 (100) | 4 (100) | 9 (75.00) | 2 (100) | 4 (57.14) | 1 (100) | 2 (100) | 2 (40) | 84.40444 |
| **Shiluvana** | Greater Tzaneen | 34021 | 79 | 7 (87.5) | 1 (100) | 4 (100) | 7 (58.33) | 2 (100) | 4 (57.14) | 1 (100) | 2 (100) | 5 (100) | 89.21889 |
| **Maake** | Greater Tzaneen | 27533 | 87 | 7 (87.5) | 1 (100) | 4 (100) | 7 (58.33) | 2 (100) | 4 (57.14) | 1 (100) | 2 (100) | 2 (40) | 82.55222 |
| **Carlotta** | Greater Tzaneen | 42884 | 71 | 7 (87.5) | 1 (100) | 4 (100) | 7 (58.33) | 2 (100) | 4 (57.14) | 1 (100) | 2 (100) | 5 (100) | 89.21889 |
| **Jamela** | Greater Tzaneen | 22738 | 72 | 7 (87.5) | 1 (100) | 4 (100) | 7 (58.33) | 2 (100) | 4 (57.14) | 1 (100) | 2 (100) | 2 (40) | 82.55222 |
| **Nkowankowa CHC** | Greater Tzaneen | 42946 | 80 | 7 (87.5) | 1 (100) | 4 (100) | 9 (75.00) | 2 (100) | 4 (57.14) | 1 (100) | 2 (100) | 2 (40) | 84.40444 |
| **Grace Mugodeni CHC** | Greater Tzaneen | 45065 | 88 | 7 (87.5) | 1 (100) | 4 (100) | 7 (58.33) | 2 (100) | 4 (57.14) | 1 (100) | 2 (100) | 5 (100) | 89.21889 |
| **Mamitwa** | Greater Tzaneen | 41503 | 62 | 7 (87.5) | 1 (100) | 4 (100) | 7 (58.33) | 2 (100) | 4 (57.14) | 1 (100) | 2 (100) | 5 (100) | 89.21889 |
| **Dr Hugo** | Greater Tzaneen | 41582 | 84 | 7 (87.5) | 1 (100) | 4 (100) | 1 (8.33) | 2 (100) | 4 (57.14) | 1 (100) | 2 (100) | 5 (100) | 83.66333 |
| **Nyavani** | Greater Tzaneen | 27234 | 71 | 7 (87.5) | 1 (100) | 4 (100) | 9 (75.00) | 2 (100) | 4 (57.14) | 1 (100) | 2 (100) | 2 (40) | 84.40444 |
| **Ooghoek** | Greater Tzaneen | 17079 | 84 | 7 (87.5) | 1 (100) | 4 (100) | 7 (58.33) | 2 (100) | 4 (57.14) | 1 (100) | 2 (100) | 5 (100) | 89.21889 |
| **Total Score (Attribute)** |  |  |  | 8 | 1 | 4 | 12 | 2 | 7 | 1 | 2 | 5 |  |
| **Average score** |  |  |  | 87.5 | 89.36 | 95.21 | 53.19 | 100 | 48.63 | 100 | 100 | 50.64 | 80.50 |
| **SCM Audit Component 95% Confidence Interval (95% CI)** |  |  |  | 87.5-  87.5 | 80.21-  98.51 | 90.71- 99.71 | 47.92-  58.46 | 100-  100 | 44.56-  52.70 | 100-  100 | 100-  100 | 43.31- 57.97 | 78.58-  82.43 |
