# Supplementary material for: Evaluating supply chain management of SARS-CoV-2 point-of-care (POC) diagnostic services in primary healthcare clinics in Mopani District, Limpopo Province, South Africa
Source: PLoS One. 2023 Jun 27;18(6):e0287477. doi: 10.1371/journal.pone.0287477 (PMC10298766; doi:10.1371/journal.pone.0287477)
Supplement: Attachment — Submitted filename: SCM and POC decision.docx [file pone.0287477.s003.docx]

Title: Kindly revise to make clear that the study was an audit of supply chain mgx at primary care centres providing POC diagnostic services.

Introduction:

Kindly rewrite focusing on the SCM systems in the country and give a background to the POC diagnostic services made available at PHC.

Methods:

1. Background on audit tool used.

2. The abstract should state that the SCM scoring was based on a questionnaire that was developed by the authors. Otherwise it gives an impression that it is some international scoring mechanism 
3. Who can perform the diagnostics?

4. Translate headcount and ideal clinic scores to language used in audit.

5. Was this one of the aims - Determining the effect of SCM on accessibility to SARS-CoV-2 POC tests and to reveal barriers and enablers of accessibility to SARS-CoV-2 diagnostic services?

6. The clinical significance of this survey is limited. I suggest that the authors “evaluate” the influence on the quality of POCT diagnostic services between different levels of storage, selection, quantification, inventory management, distribution, and human resource capacity.

7. Why and how were the participants trained to conduct the audit?

8. Please use an appropriate format for reporting a clinical audit, for example <https://www.nice.org.uk/media/default/sharedlearning/721_clinicalaudit_report_729_re-auditcopd.pdf>

<https://cpd.osteopathy.org.uk/resources/audit-report-template-2/>

Results:

1. Rewrite the results section and transform texts to figures/ graphics.

2. In addition to descriptive analyses, please perform inferential analyses.

3. The presentation of this section need to be improved.

Discussion:

1. Highlight SCM importance to diagnostics in that access is usually impacted by supply chain parameters.
2. The conclusion section is not strong enough. It is important to mention why there is a lot of variation in the districts in the audit parameters? 
3. It should be mentioned that the limitation of this study is that only one area was surveyed. Whether these SCM issues are related to this area (as it is a rural area) or it is a bigger issue, we still do not know.

Conclusion:

Please rewrite the conclusion; recommendations should be a subheading in the “Discussion” section.

Additional comment:

Please have the manuscript edited by a native English speaker.
