# Supplementary material for: Evaluating supply chain management of SARS-CoV-2 point-of-care (POC) diagnostic services in primary healthcare clinics in Mopani District, Limpopo Province, South Africa
Source: PLoS One. 2023 Jun 27;18(6):e0287477. doi: 10.1371/journal.pone.0287477 (PMC10298766; doi:10.1371/journal.pone.0287477)
Supplement: Attachment — Submitted filename: Address reviewers and editors comments.docx [file pone.0287477.s005.docx]

Dear Editor,

Thank you for providing us an opportunity to revise and re-submit a revised draft of out manuscript titled *“Evaluating supply chain management of SARS-CoV-2 point-of-care (POC) diagnostic services in primary healthcare clinics in Mopani District, Limpopo Province, South Africa”* to *Plos One.*

We appreciate the time and the effort that you and the reviewers have dedicated to providing your valuable feedback on my manuscript. We grateful to the reviewers for their insightful comments on my paper. We have been able to incorporate changes to reflect most of the suggestions provided by the reviewers. All changes are highlighted in yellow throughout the manuscript.

Below is a table presenting our point-by-point response to the reviewer’s comments and concerns

**Addressing Reviewer’s & Editor’s comments**

| **Reviewer comments** | **Author’s comments** | **Page & line number** |
| --- | --- | --- |
| 1. Title: Kindly revise to make clear that the study was an audit of supply chain mgx at primary care centres providing POC diagnostic services. | The title has been revised. It now reads:  Evaluating supply chain management of SARS-CoV-2 point-of-care (POC) diagnostic services in primary healthcare clinics in Mopani District, Limpopo Province, South Africa | Page number 1  Line number 2-4 |
| 2. Introduction: Kindly rewrite focusing on the SCM systems in the country and give a background to the POC diagnostic services made available at PHC. | The introduction has been revised and it now focuses on SCM systems in the country and a background of the POC diagnostic services. | Page number 3-5  Line number 51-100 |
| 3. Methods |  |  |
| 3.1. Background on audit tool used. | Please note the authors developed an audit tool and this statement is included in the manuscript:  The audit tool used (S1 Fig1) in this study was developed based on the WHO guidelines for improving the quality of POC testing (25) and the Management Sciences for Health (MSH) diagnostic SCM guideline (26). We focused solely on aspects related to SCM and used these documents to evaluate the challenges that resource-limited settings face in accessing appropriate, quality-assured, and adequate POC diagnostics, as well as to identify potential strategies for addressing these challenges. | Page number 7-8  Line number 139-145 |
| 3.2. The abstract should state that the SCM scoring was based on a questionnaire that was developed by the authors. Otherwise it gives an impression that it is some international scoring mechanism | A sentence has been inserted that reads:  One participant per clinic completed an audit tool developed by the authors with guidance from the World Health Organization and the Management Sciences for Health guidelines. The audit tool evaluated the following SCM parameters: selection, quantification, storage, procurement, quality assurance, distribution, redistribution, inventory management, and human resource capacity. | Page number 2  Line number 29-34 |
| 3.3. Who can perform the diagnostics? | Please note that this research did not involve performing of the diagnostics, but we evaluated the SCM of the diagnostic tests that are currently offered at the PHC clinic and performed by healthcare workers. |  |
| 3.4. Translate headcount and ideal clinic scores to language used in audit. | Definition of the ideal clinic scores and the headcount are given under the heading “Audit tool”. The statement reads:    The audit tool included clinic characteristics such as: i) annual headcount, or number of patients who present to the PHC clinic regardless of the health service provided; and ii) the ideal clinic score, where an ideal clinic is defined as a clinic with good infrastructure, adequate staff, adequate medicine and supplies, good administrative processes, and sufficient adequate bulk supplies (12). | Page number 8  Line numbers 145-149 |
| 3.5. Was this one of the aims - Determining the effect of SCM on accessibility to SARS-CoV-2 POC tests and to reveal barriers and enablers of accessibility to SARS-CoV-2 diagnostic services? | The main objective of this study was to evaluate the SCM for SARS-CoV-2 POC diagnostic services in resource-limited settings. This evaluation was aimed at determining the effect of SCM on accessibility to SARS-CoV-2 POC tests and to identify barriers and enablers of accessibility to SARS-CoV-2 diagnostic services. | Page numbers 5  Line numbers 97-100 |
| 3.6. The clinical significance of this survey is limited. I suggest that the authors “evaluate” the influence on the quality of POCT diagnostic services between different levels of storage, selection, quantification, inventory management, distribution, and human resource capacity. | Thank you for the comment however this not aligned with the current aim of the study. We take note of the suggestion and we will include this in the planned future research. |  |
| 3.7. Why and how were the participants trained to conduct the audit? | The audit questionnaire was administered by the PI after presenting a summary of the study and obtaining consent from participants. The participants were not trained to conduct the audit, they responded to the PI’s questions which were drawn from the audit questionnaire. | Page number 8  Line numbers 153-154 |
| 3.8. Please use an appropriate format for reporting a clinical audit, for example https://www.nice.org.uk/media/default/sharedlearning/721_clinicalaudit_report_729_re-auditcopd.pdf   https://cpd.osteopathy.org.uk/resources/audit-report-template-2/ | Thanks for your suggestion. Please note that this was not a clinical audit but an audit evaluating SCM of the POC diagnostic test. |  |
| 4. Results |  |  |
| 4.1. Rewrite the results section and transform texts to figures/ graphics. | The results section is organised into different sections with graphics to visualise the data summarised in the texts. | Page numbers 10-23  Line numbers 203-391 |
| 4.2. In addition to descriptive analyses, please perform inferential analyses. | We included inferential analyses in terms of 95% confidence intervals for the component scores. We also included a one-way anova test to compare the scores between the 5 sub-districts and we provided the p-values. | Page number 15-17  Line numbers 258-261 |
| 4.3. The presentation of this section needs to be improved. | The results section is organised into different sections with graphics to visualise the data summarised in the texts. To add more clarity on the SCM audit components we added a description of the results in that section to help the reader understand what is being presented. The statement reads:  The SCM component scores are summarized in Figure 3. In this section we describe the barriers and enablers of each component individually. | Page numbers 10-23  Line numbers 203-391 |
| 5. Discussion |  |  |
| 5.1. Highlight SCM importance to diagnostics in that access is usually impacted by supply chain parameters. | The discussion section has been revised and now highlights the importance of SCM parameters in accessibility of POC tests. | Page numbers 24  Line numbers 400-403 |
| 5.2. The conclusion section is not strong enough. It is important to mention why there is a lot of variation in the districts in the audit parameters? | The conclusion has been revised and now mentions why there are variations within the sub-districts. | Pages 26  Line numbers 471-473 |
| 5.3. It should be mentioned that the limitation of this study is that only one area was surveyed. Whether these SCM issues are related to this area (as it is a rural area) or it is a bigger issue, we still do not know. | The following statement has been added under the discussion section:  Another limitation was that this audit was only conducted in one district, which doesn’t allow comparison with other areas. | Pages 25  Line numbers 441-442 |
| 6. Conclusion   Please rewrite the conclusion; recommendations should be a subheading in the “Discussion” section. | The conclusion has been revised.  A sub-heading “recommendations” added in the “Discussion” section. | Pages 26-27  Line numbers 466-76  Pages 25-26  Line numbers 443-465 |
| 7. Additional comment:   Please have the manuscript edited by a native English speaker. | The manuscript has been audited by a native English speaker | Whole document |
| 8. We note that Figure 1 in your submission contain map image which may be copyrighted. | Please note that Figure 1 is not copyrighted however it has been created by the first author on ArcGIS as a graphic representation of the study setting.  The figure legend has been revised:  Fig 1. Geographic location and distribution of the audited primary healthcare clinics in the Mopani district, Limpopo province, South Africa. Image created by authors on ESRI’s ArcGIS 10.8.2; no copyrighted material was used. | Page number 7  Line numbers 121-123 |
